# Conflict in the Indian Kashmir Valley I: exposure to violence

**DOI:** 10.1186/1752-1505-2-10

**Published:** 2008-10-14

**Authors:** Kaz de Jong, Nathan Ford, Saskia van de Kam, Kamalini Lokuge, Silke Fromm, Renate van Galen, Brigg Reilley, Rolf Kleber

**Affiliations:** 1 Médecins Sans Frontières, Plantage Middenlaan 14, 1018 DD Amsterdam, the Netherlands; 2 Faculty of Health Sciences, Simon Fraser University, Vancouver, Canada; 3 Department of Clinical Psychology, Utrecht University, the Netherlands

## Abstract

**Background:**

India and Pakistan have disputed ownership of the Kashmir Valley region for many years, resulting in several conflicts since the end of partition in 1947. Very little is known about the prevalence of violence and insecurity in this population.

**Methods:**

We undertook a two-stage cluster household survey in two districts (30 villages) of the Indian part of Kashmir to assess experiences with violence and mental health status among the conflict-affected Kashmiri population. The article presents our findings for confrontations with violence. Data were collected for recent events (last 3 months) and those occurring since the start of the conflict. Informed consent was obtained for all interviews.

**Results:**

510 interviews were completed. Respondents reported frequent direct confrontations with violence since the start of conflict, including exposure to crossfire (85.7%), round up raids (82.7%), the witnessing of torture (66.9%), rape (13.3%), and self-experience of forced labour (33.7%), arrests/kidnapping (16.9%), torture (12.9%), and sexual violence (11.6%). Males reported more confrontations with violence than females, and had an increased likelihood of having directly experienced physical/mental maltreatment (OR 3.9, CI: 2.7–5.7), violation of their modesty (OR 3.6, CI: 1.9–6.8) and injury (OR 3.5, CI: 1.4–8.7). Males also had high odds of self-being arrested/kidnapped (OR 8.0, CI: 4.1–15.5).

**Conclusion:**

The civilian population in Kashmir is exposed to high levels of violence, as demonstrated by the high frequency of deliberate events as detention, hostage, and torture. The reported violence may result in substantial health, including mental health problems. Males reported significantly more confrontations with almost all violent events; this can be explained by higher participation in outdoor activities.

## Background

The British rule over Jammu and Kashmir terminated in 1947. During partition, the Kashmiri population – the majority of whom is Muslim – was promised a choice of joining either India or Pakistan through a popular vote but this plebiscite never took place. Instead, partition was the start of a long history of conflict affecting the roughly 8 million inhabitants of Kashmir [1]. Both India and Pakistan have made control of a unified Kashmir an essential cornerstone of their national identities and have fought several wars between 1947 and 2002 on this issue. The ceasefire line between Pakistan and India, named the "Line of Control" in 1972, still exists today, separating this territory of around 2.2 million square kilometres into three parts. India controls the largest part, with the rest governed by Pakistan and China [1].

Up to twenty years ago the conflict was mainly an interstate affair between Pakistan and India, but in 1988 Kashmiri militants started a liberation movement. The low level war ('militancy') between the liberation movement and the Indian army spiralled into a cycle of armed conflicts with the civilian population caught between the fighting parties. Officially, 20,000 have died and 4, 000 have disappeared since the start of the militancy – in 2004 alone, 1587 militancy incidents and 1263 deaths including 479 civilians were officially recorded [1] – however, according to other sources these figures are substantially higher [2]. The conflict has also led to displacement of Kashmiri Hindu or Pundits and Muslims from the Kashmir Valley.

Violence affects nearly everybody living in Kashmir. A recent population survey [3] found a lifetime prevalence of traumatic events of 59% among the inhabitants of four districts of the Indian part of Kashmir. The most frequent traumatic events encountered were: firing and explosions (81%) and exposure to combat zones (74%). Traumatic events and the way people cope with them have a crucial role in the development of psychological distress and pathology such as anxiety disorders (including Post Traumatic Stress Disorder) and major depressive disorder [4]. Very little is known about the psychological impact of the insecurity on the Kashmiri population.

To assist in determining the future direction of medical humanitarian assistance in the Indian part of Kashmir, Médecins Sans Frontières (MSF) undertook a quantitative population survey to assess the frequency and nature of violence confronted by the population living in the Indian part of Kashmir and its impact on psychological health and socio-economic functioning. This paper presents the main findings related to exposure, witnessing and self-experiencing of violence. Data on the mental health impact of the conflict is presented in a separate paper [5]

## Methods

The study design was based on a methodology previously used in other conflict settings [6]. A two-stage cluster design was executed in two districts in the Indian part of Kashmir (Kupwara and Badgam). These districts were chosen because MSF intended to start working there, an operational decision based on anecdotal evidence of mental health problems among populations living in these areas. The districts have a combined population of 145,000 residents living in 101 villages (3750 square kilometres). The predominantly Muslim, rural and indigenous population of these districts do not differ from other districts in Kashmir except for the capital, Srinagar. Both districts are close to the Line of Control and have experienced high numbers of violent incidents, although to what degree the level of violence differs from other districts is unknown due to lack of reliable information.

For the calculation of sample size we assumed a prevalence of trauma-related psychological problems of 20% [4], and using a precision of 5% (confidence interval 95%) and a design effect of 2, the minimum sample size was estimated at 492. A two-stage cluster sampling design was used to cover 30 villages, resulting in 17 randomly selected households per village. Research teams started at the centre of the village, spun a bottle, and began the interviews according to the direction in which the bottle pointed. The first encountered household was selected, after which the next household in the same direction was approached. Within the household the participant was also selected randomly.

### Ethics and interview procedures

The survey was conducted over a period of eleven weeks, from 4 June 2005 to 16 August 2005 in Badgam and from 4 July 2005 to 18 August 2005 in Kupwara. The informed consent procedure consisted of two steps. In the first step the head or most senior adult present in each selected household was asked permission to interview a person over the age of 18 years. The purpose of study, guarantees of anonymity and confidentiality, the use of data (including public dissemination and scientific publication), and the possibility to withdraw from interview at any time was explained. It was made clear that no (financial) compensation was given. Written consent was then sought. The head of household assisted the interviewer in making a list of all household members and from this list one person (the respondent) above 18 years of age was selected randomly. If the selected person was not at home, another person in the household (>18 years) was selected. Step two of the interview process consisted of repeating the above introduction to the potential participant. Once written consent was given, the interview was conducted.

The survey team consisted of four senior national and expatriate staff that supervised 20 trained local interviewers. Interviews were done in pairs, each pair conducting two to three interviews each day. Each team consisted of both male and female interviewers and respondents could choose who did the interview. The average time for interviewing was 50–60 minutes. The interviewers were recruited from Srinagar University Department of Psychology and Sociology and received a salary for their work. Teams stopped their activities at any moment if they were worried about their own safety or that of the population or if they judged their activities to be counterproductive to the program (for instance, when security incidents such as strikes or 'Hartals' occurred, forcing the survey team to postpone the survey).

Interviewing people on traumatic experiences carries a risk of contributing to psychological distress of both interviewee and interviewer. To respond to this, one experienced counsellor supervised each survey team to give immediate (technical or emotional) support if required. Also, referral to MSF operated counselling centres in another location was offered to all interviewees and interviewers (although none were referred).

To manage potential overwhelming emotions among the interviewer, staff training was given in communication and handling of difficult or upsetting situations. Staff were debriefed daily for both technical and emotional issues. For those interviewers who were overwhelmed or needed follow-up support counselling services were available.

The study received ethics approval from MSF's independent Ethical Review Board.

#### Instruments

The survey questionnaire was based on previous formats used in similar studies elsewhere [6] and focussed on the following four subjects: baseline demographics, confrontation with and consequences of violence, mental health, and sources of support. This paper focuses on the first two issues. Tools to assess mental health, and sources of support are described in a second paper [5].

We assessed confrontation with violence both since the beginning of the conflict and in the three months preceding the survey. Proximity to violence was defined as either exposure ('Being in the vicinity of a violent event but not witnessing or self-experiencing'), witnessing ('Witnessing an event so close it could have happened to you or you were forced to see it'), or self-experience ('The event happened to you'). Violence categories were based on a review of violent incidents as reported in newspaper articles (such as Kashmir Affairs, Greater Kashmir, and Jammu Kashmir) of the past two years and consultation with national staff. We used rape in the witnessing section and a broader concept of 'violation of modesty' in the self-experience section because national staff felt that interviewees would feel more comfortable with this term. Violation of modesty is the local equivalent for sexual violence and includes inappropriate touching, in accordance with the WHO's definition of sexual violence [8].

The survey was translated from English to Urdu and phonetic Kashmiri, then back-translated from Urdu and phonetic Kashmiri to English using a different translator. After revisions, the questionnaire was piloted in a community close to Srinagar. For the definition of the start of the conflict (1989), the definition of torture ('Unbearable physical pain deliberately inflicted by others who have complete control'), maltreatment ('cruel and inhumane treatment'), and round-up raids the local population and national staff were consulted. Examples of physical and mental maltreatment such as 'Being kicked at checkpoints', and 'For body searching males being forced to undress in front of their family' were discussed among interviewers, as were forced labour and violation of modesty.

#### Analysis

Data entry was standardised and checked by supervisors. As an additional control, 5% of the forms were randomly checked. Data were entered in an EXCEL program spreadsheet and exported into EPIINFO-2002 for analysis. Previous studies have consistently shown gender to be a risk factor for developing psychological problems (most notably post-traumatic stress disorder) after exposure to traumatic events [9,10]. Analysis of our data also revealed gender as a confounder for many variables. Therefore we stratified results by gender (see Tables).

## Results

510 of 548 (93%) interviews were completed. Reasons for refusal to participate (25) and stopping the interview (13) included: lack of time, distrust, and being emotionally upset. The survey was interrupted for 10 days due to security incidents and official strikes. The number of incidents that occurred was not considered exceptional for the area.

The average age of respondents was 37.7 years (range 17–90) with an equal gender distribution (males = 53%; 270; p > 0.05), similar to general statistics on household composition in the district (53.4% males) [19]. Respondents reported having an average household of nine persons (8.94; males: 2425, females: 2126). Nearly all respondents were originally from the Kashmir area (498; 97.6%). The majority of respondents were married (75.2%; 379) and half (52.6%; 266) had no formal schooling. A quarter of respondents (24.9%; 127) reported high or total dependence on financial/material assistance from the authorities or from charity.

Confrontation with violence was reported both in the past (since 1989) and more recently (three months prior to the survey). Exposure to crossfire (Table 1) was commonly reported both since the start of conflict (61.4%; 313) and in the previous three months (14.3%; 73). Over eight in ten people (82.7%; 422) were exposed to round up raids, including in the previous 3 months (9.8%; 50).

**Table 1:** Exposure to violence by gender (n = 510)

**Exposure**	**Since 1989**	
Crossfire	85.7% (437)	
*Since 1989 *≥ 5×	61.4% (313)	
*Past 3 months*	14.3% (73)	
*Males*	*88.1%*	(P < .119; OR 1.5, CI: 0.9–2.5)
*Females*	*82.9%*	
Round-up raids	82.7% (422)	
*Since 1989 *≥ 5×	61.6% (314)	
*Past 3 months*	9.8% (50)	
*Males*	*86.3%*	(P < .003; OR 1.7, CI: 1.1–2.7)
*Females*	*78.8%*	
Explosion of mines/grenades	64.5% (329)	
*Since 1989 *≥ 5×	37.3% (190)	
*Past 3 months*	12.0% (61)	
*Males*	*71.5%*	(P < .001; OR 1..9, CI: 1.3–2.8)
*Females*	*56.7%*	
Damage to property	39.0% (199)	
*Since 1989 *≥ 5×	17.3% (88)	
*Past 3 months*	2.8% (14)	
*Males*	*45.2%*	(P < .003; OR 1.7, CI: 1.2–2.5)
*Females*	*32.1%*	
Burning of houses	26.3% (134)	
*Since 1989 *≥ 5×	13.1% (67)	
*Past 3 months*	2.0% (10)	
*Males*	*31.3%*	(P < .011; OR 1.7, CI: 1.1–2.0)
*Females*	*20.8%*	

Note: P Chi square Yates corrected unless indicated differently

Table 2 reports the incidence of witnessed events. Almost three quarters of people (73.3%; 374) witnessed physical or mental mistreatment, half (50%; 255) having witnessed such events on multiple occasions. Over two-third of people (66.9%; 341) witnessed someone being tortured, often on multiple occasions (38.4%; 196), including during the three months prior to the survey (13.5%; 69). Forty per cent of people (322) saw someone being killed, including in the three months prior to the survey (12.6%; 64). Over one in ten people (13.3%; 68) had witnessed rape; sometimes on multiple occasions (5.1%; 26) including in the three previous months (2.2%; 11).

**Table 2:** Witnessing violence by gender (n = 510)

**Witness**	*Since 1989*	
Persons arrested	75.5% (385)	
*Since 1989 *≥ 5×	52.9% (270)	
*Past 3 months*	12.8% (65)	
*Males*	*83.7%*	(P < .000; OR 2.6, CI: 1.7–4.0)
*Females*	*66.3%*	
Physical/mental mistreatment	73.3% (374)	
*Since 1989 *≥ 5×	50% (255)	
*Past 3 months*	9.8% (50)	
*Males*	*83%*	(P < .000; OR 2.9, CI: 1.9–4.4)
*Females*	*62.5%*	
Persons tortured	66.9% (341)	
*Since 1989 *≥ 5×	38.4% (196)	
*Past 3 months*	13.5% (69)	
*Males*	*74.8%*	(P < .000; OR 2.2, CI: 1.5–3.1)
*Females*	*57.9%*	
Persons wounded	63.1% (322)	
*Since 1989 *≥ 5×	35.5% (181)	
*Past 3 months*	14.5% (74)	
*Males*	*73%*	(P < .000; OR 2.5, CI: 1.7–3.6)
*Females*	*52.1%*	
Persons killed	40.0% (204)	
*Since 1989 *≥ 5×	17.3% (88)	
*Past 3 months*	12.6% (64)	
*Males*	*44.1%*	(P < .057; OR 1.4, CI: 1.0–2.1)*
*Females*	*35.4%*	
Hear of cases of rape	63.9% (326)	
*Since 1989 *≥ 5×	38.2% (195)	
*Past 3 months*	10.8% (55)	
*Males*	*75.2%*	(P < .000; OR 2.9, CI: 2.0–4.2)
*Females*	*51.3%*	
Seen Rape	13.3% (68)	
*Since 1989 *≥ 5×	5.1% (26)	
*Past 3 months*	2.2% (11)	
*Males*	*17.4%*	(P < .006; OR 2.2, CI: 1.3–3.8)
*Females*	*8.8%*	

*Yates corrected

Almost half of people interviewed (44.1%; 225) reported being physically or mentally mistreated themselves (self-experience, Table 3) since the start of the conflict, many repeatedly (18.6%; 95). A third (33.7%; 172) had undergone forced labour, the majority of these (55%; 95) on multiple occasions. One in six people (16.9%; 86) had been detained or held hostage, and the majority of these reported being tortured (76.7%; 66; n = 86). More than one in ten (11.6%; 59) had been subjected to a violation of modesty (sexual violence), many repeatedly (47%; 28).

**Table 3:** Self-experienced violence by gender (n = 510)

**Self-experienced**	**Since 1989**	
Physically or mentally mistreated	44.1% (225)	
*Since 1989 *≥ 5×	18.6% (95)	
*Past 3 months*	3.9% (20)	
*Males*	*59.3%*	(P < .000; OR 3.9, CI: 2.7–5.7)
*Females*	*27.1%*	
Forced labour	33.7% (172)	
*Since 1989 *≥ 5×	18.2% (95)	
*Past 3 months*	2.0% (10)	
*Males*	*46.7%*	(P < .000; OR 3.7, CI: 2.5–5.5)
*Females*	*19.2%*	
Forced to house any of the parties	18.4% (94)	
*Since 1989 *≥ 5×	7.5% (38)	
*Past 3 months*	1.2% (6)	
*Males*	*24.8%*	(P < .000; OR 2.6, CI: 1.6–4.2)
*Females*	*11.3%*	
Have you been arrested/kidnapped?	16.9% (86)	
*Since 1989 *≥ 5×	2.2% (11)	
*Past 3 months*	0.6% (2)	
*Males*	*27.8%*	(P < .000; OR 8.0, CI: 4.1–15.5)
*Females*	*4.6%*	
Tortured during detention/hostage	*76.7% (66)*	
*Since 1989 *≥ 5×	15.1% (13)	
*Past 3 months*	1.2% (1)	
*Males*	*78.7%*	(P < .472; OR 2.1, CI: 0.6–8.1)
*Females*	*63.6%*	
Violation of modesty	11.6% (59)	
*Since 1989 *≥ 5×	5.5% (28)	
*Past 3 months*	1.6% (8)	
*Males*	*17. 0%*	(P < .000; OR 3.6, CI: 1.9–6.8)
*Females*	*5.4%*	
Injury	5.5% (28)	
*Since 1989 *≥ 5×	0.4% (2)	
*Past 3 months*	0.4% (2)	
*Males*	*8.1%*	(P < .009; OR 3.5, CI: 1.4–8.7)
*Females*	*2.5%*	

In all categories, but particularly for witnessing and self-experiencing, males reported significantly more confrontations with violence. Males had an increased likelihood of being subjected to physical/mental maltreatment (OR 3.9, CI: 2.7–5.7), forced labour (OR 3.7, CI: 2.5–5.5), violation of modesty (OR 3.6, CI: 1.9–6.8) and injury (OR 3.5, CI: 1.4–8.7), and had a higher odds of being arrested/kidnapped (OR 8.0, CI: 4.1–15.5).

## Discussion

This paper presents findings related to confrontation with violence among the conflict-affected Kashmiri population. We did not assess who was responsible for the violence because it was not relevant for our medical needs assessment. We found a high exposure to violence (being in the vicinity but not witnessing or self-experiencing) among the civilian participants in our survey, reflecting a pervasive climate of violence in which the population is living. The frequency of exposure to violence on multiple occasions (>5 times) since the start of the conflict (Table 1) is high and comparable to a study from Afghanistan reporting that 62.0% of the participants experienced at least 4 traumatic events during the previous 10 years [11]. The violence in Kashmir, which began in 1989, was noted up until the date of the survey (August 2005).

High levels of confrontation with violence have been reported in another recent study from Kashmir. In this study, no substantial differences between males (59.51%) and females (57.39%) were found for lifetime prevalence of traumatic experiences. [3] The study also lacks details of specific violence-related events, and does not differentiate between exposure, witnessing and self-experiencing. Our study found the number of confrontations with violence was significantly higher for males, particular for events such as witnessing persons being arrested, maltreated, tortured, or wounded, or hearing about and witnessing rape. Males also 'self-experienced' more violence such as maltreatment, forced labour and forced housing of one of the warring parties. Our findings are in line with a recent meta-analysis that showed a significant higher confrontation with violence for males than for females in other contexts [10], and may be due to the socio-economic activities of males that mean they spend a significant amount of time outdoors whereas women tend to spend more time in the home.

The high level of people reporting being tortured while detained or taken hostage is a particular concern, indicating that the violence against civilians is not simply circumstantial. We used "violation of modesty" as the local equivalent for sexual violence [8]. The fact that men reported this more frequently than women that is surprising: in most studies females are more frequently subjected to sexual violence, partly because males are reluctant to report sexual violence [12,13]. People may have misunderstood the concept 'violation of modesty' despite extensive piloting and consultation with national staff and counsellors many of whom are males themselves. The high frequency of violation of modesty reported by males might be partly explained by the high frequency of body searching to which Kashmiri men are subjected. Whether the body searching is perceived as inappropriate touching (part of the definition of 'Modesty violation') or the way of touching is remains unclear. A substantial number of males that reported being detained or taken hostage also reported being tortured (77%), and this may also have been understood as a 'violation of modesty'.

### Potential limitations

The completion rate of the survey was good (93%), and the design was adapted to the purpose and the context. However, there are a number of potential limitations. First, there is a possible selection bias in the fact that only people who were home during the time of the survey were interviewed. This methodology was deemed necessary for security reasons. The selection of one person per household may lead to a bias as individuals in large households are under represented. However, we do not think this bias influenced our findings since the overall household size in our sample was large (9). Second, retrospective study designs are subject to recall bias, and we cannot exclude recall bias in the participants' answers on confrontations with violence. However, a recent study [14] has demonstrated that conflict-affected populations remain consistent in reporting on major traumatic events over time. Finally, there may have been confusion over definitions of terms such as violation of modesty as discussed above.

## Conclusion

This survey aimed to determine exposure to violence and mental health impact as part of a routine programme assessment. We found that the Kashmiri population is confronted with high levels of violence committed by all parties to the conflict, with potentially substantial implications for mental health. This confrontation with violent events is not simply an environmental effect of living in a conflict-affected area, as demonstrated by the high frequency of deliberate events as detention, hostage, and torture. The conflict continues with no end in sight, with civilian deaths reported as this article goes to print [15].

## Conflicts of interests

The authors declare that they have no competing interests.

## Authors' contributions

KJ designed and co-ordinated the study and wrote the first draft of the paper. NF supported the conceptual framing of the findings, assisted with the analysis, and led subsequent drafts. SK and KL provided statistical support for the design and analysis, and helped with the writing of the paper. SF, RG and BR oversaw the implementation of the survey, managed data collection in the field, and contributed to the writing of the paper. RK provided conceptual oversight and contributed to the writing of the paper.
